# Refining clinical features and therapeutic options of new daily persistent headache: a retrospective study of 63 patients in India

**DOI:** 10.1007/s10194-012-0461-6

**Published:** 2012-05-27

**Authors:** Sanjay Prakash, Samir Saini, Kaushikkumar Ramanlal Rana, Pinaki Mahato

**Affiliations:** 1 Department of Neurology, Medical College, SSG Hospital, O-19, Doctor’s Quarter, Jail Road, Baroda, Gujarat India 390001; 2 Department of Medicine, Medical College, Baroda, Gujarat India 390001

**Keywords:** Headache, New daily persistent headache, Chronic daily headache

## Abstract

The aim of this retrospective study was to provide data on the clinical features and treatment outcomes of patients with NDPH (fulfilling Kung et al.’s criteria). A total of 63 patients were observed during a 5-yr period (2007–2012). More than one-third (35 %) patients had migrainous features; 65 % patients fulfilled the ICHD-II criteria. Both groups were similar in most clinical and epidemiological features. However, migrainous features were more common in patients with a prior history of episodic migraine (though statistically not significant). After a median follow-up of 9 months, 37 % patients showed “excellent” response (no or less than 1 headache per month). Another 30 % patients had “good” response (>50 % reduction in headache frequency or days per month). Excellent response was more in patients with a history of less than 6 months duration (statistically not significant). Patients with a recognized trigger showed better prognosis. Response was better in patients who received intravenous therapy of methyl prednisolone and sodium valproate. We suggest prospective and controlled studies to confirm our observations.

## Introduction

More than 25 years have passed since the first description of new daily persistent headache (NDPH) by Vanast [1]. However, its clinical features and natural history are still being determined [2, 3]. The International Headache Society (IHS) proposed a diagnostic criterion in the second edition of the International Classification of Headache Disorders (ICHD-II) [4]. However, a few authors suggest that current ICHD-II criteria for NDPH are too restrictive, and a few new criteria have been suggested in the recent past in the literature [2, 3]. The main characteristic feature of NDPH is daily and unremitting headache from the onset or from less than 3 days from the onset [4]. The ICHD-II criterion for NDPH resembles daily form of chronic tension-type headache (CTTH) that begins abruptly [4].

The main controversy in the diagnostic criteria is regarding the presence of migrainous features [5]. ICHD-II acknowledges only one of photophobia, phonophobia, or mild nausea in primary NDPH. However, most other migrainous features are against the diagnosis of NDPH. Unilateral head pain, throbbing headache, severe intensity, exacerbations by physical activities, moderate to severe nausea, and vomiting are not the features of NDPH. However, most studies suggest that migrainous features may be the part of the clinical spectrum of NDPH or a clinical sub form of NDPH exists [6–9]. Initially, NDPH was considered as a “benign or self-limiting” form of headache. But, recent observations suggest that it may be the most treatment refractory of all primary headache disorders.

In this retrospective study, we studied a group of consecutive patients who fulfilled the Kung et al.’s revised criteria for NDPH. We mainly looked for the clinical features and treatment responsiveness in these patients.

## Materials and methods

This study was conducted as a retrospective chart review of patients seen in Neurology Department in our institute (a tertiary centre) from January 2007 to February 2012. The study constitutes a consecutive series of patients who were diagnosed as having NDPH. NDPH was diagnosed according to the revised ICHD-II criteria (Kung et al.’s criteria). Most recent studies on NDPH have used this criterion. Kung et al.’s criteria include only criteria A and B of ICHD-II. It reads as (A) headache more than 3 months, and (B) headache is daily and unremitting from onset or less than 3 days from onset. Patients with a history of episodic migraine or episodic tension-type headache (≤1 attack/month) were included in the study.

We reviewed each individual’s chart with the diagnosis of NDPH. If the chart was not complete then the patient was interviewed by phone to retrieve the missing information. Age and duration of illness were determined as of the date of first visit. The patients who did not have headache duration of >3 months at the time of first visit were excluded from the study. We also recorded the medications used and their responses. As there are no well-defined guidelines in the literature for the treatment of NDPH, the treatment strategy was not standardized and the treatment plans were made at the discretion of the treating physician. The follow-up clinical response was rated by the treating physician as: Excellent (no or less than 1 headache per month), Good (>50 % reduction in headache frequency or days per month), Fair (<50 % reduction in headache frequency or days per month), and Poor (Minimal or no response).

Exclusion criteria included (a) a possible secondary NDPH; (b) patients who were never subjected for neuroimaging, as we did not rule out the possibility of secondary NDPH in these patients; and (c) a follow-up of <3-months duration.

The majority of patients were seen and examined by a neurologist who has a special interest in headache disorders. A neurological examination including fundoscopy was performed on all patients. The study did not require approval by the local ethics committee as per the local regulations for retrospective observation. Patients who reported earlier to our institute were included to complete the data.

Data are presented as percentage or as arithmetic mean with SD. Student’s *t* test was used to compare the continuous data. The Fisher-exact test was used for categorical data. All *p* values were two-tailed, and a *p* value <0.05 was defined as statistically significant.

## Results

We identified 69 patients with a diagnosis of NDPH. Three patients were excluded because of the possibility of secondary NDPH (2 patients: benign intracranial hypertension, 1: brain tuberculoma). Two patients were excluded as they were never subjected to cranial neuroimaging. One more patient was excluded because of incomplete follow-up (<3 months). Finally 63 patients were identified who fulfilled the Kung et al.’s criteria for NDPH. Forty-one patients (65 %) fulfilled the IHS criteria for HC (NDPH-ICHD). Twenty-two patients (35 %) had prominent migrainous features. We labeled this group as NDPH-mf (migrainous features) (as suggested by Robbins et al.’s). Epidemiological and clinical features are summarized in Table 1. Patients subgroup (NDPH-ICHD and NDPH-mf) were compared using Fisher exact (2-tailed).

**Table 1:** Epidemiological profiles and clinical features in NDPH (Kung et al.’s), NDPH (ICHD-II) and NDPH (mf)

	NDPH (Kung et al.’s), *n* (%)	NDPH (ICHD-II), *n* (%)	NDPH-mf, *n* (%)	*p* value
No. of patients	63	41 (65 %)	22 (35 %)	
Age (years) (mean, range)	36.8 ± 12.8, 18–68	35.2 ± 11.5, 18–58	39.9 ± 14.1, 20–68	0.1585
Gender (female)	36 (57 %)	24 (59)	12 (55 %)	0.7946
Duration of illness				
Mean (SD)	27.5 ± 19.6 months	25.14 ± 19.4 months	29.7 ± 19.9 months	0.3774
Range	3–72 months	4–60 months	3–60 months	
Past history of TTH	18 (29)	13 (32)	5 (23)	0.5642
Past history of migraine	16 (25)	9 (22)	7 (32)	0.5446
Recalling onset day	21 (33)	14 (34)	7 (32)	1.000
Unilateral pain	11 (17)	4 (9)	7 (32)	0.0394^*^
Daily pain	63 (100)	41 (100)	22 (100)	1.000
Pain free period in a day	7 (11)	5 (12)	2 (9)	1.000
Type of pain				
Non-throbbing	63 (100)	41 (100)	22 (100)	1.000
Throbbing	32 (51)	18 (54)	14 (64)	0.1877
Aggravation by physical activities	17 (27)	10 (24)	7 (32)	0.5619
Hampering routine activities/professional work	5 (8)	3 (7)	2 (9)	1.000
Associated features				
Nausea	31 (49)	12 (29)	19 (86)	0.0001^*^
Vomiting	3 (5)	0 (0)	3 (14)	0.0388^*^
Photophobia	21 (33)	7 (17)	14 (64)	0.0005^*^
Phonophobia	12 (19)	3 (7)	9 (41)	0.0025^*^
Autonomic symptoms				
Conjunctival-injection/tearing	9 (14)	4 (9)	5 (23)	0.2564
Depression (self-reported)	12 (19)	8 (20)	4 (18)	1.000
Anxiety (self-reported)	10 (16)	7 (17)	3 (14)	1.000
Triggering factors				
None	29 (46)	19 (46)	10 (45)	1.000
Infection	18 (29)	10 (24)	8 (36)	0.3850
Injury	7 (11)	4 (9)	3 (14)	0.6871
Stress	5 (8)	5 (12)	–	0.1530
Surgery	3 (5)	2 (5)	1 (5)	1.000
Post-partum	1 (2)	1 (2)	–	1.000
Medication Overused	8 (13)	5 (12)	3 (14)	1.000

*NDPH (ICHD-II)* new daily persistent headache according to second edition of the International Classification of Headache Disorders, *NDPH-mf* new daily persistent headache with prominent migrainous features, *TTH* tension-type headache

### Epidemiological and clinical features (Table 1)

Age of onset ranged from 18 to 68 years (mean 36.8 ± 12.8 years). 57 % patients were female. The average time between the onset of the symptoms and the first visit to our institute was 27.5 months (± 19.6 months). Twenty-seven patients (43 %) had headache duration of more than 2 years at the time of first consultation and 12 patients (19 %) had duration of ≤6 months (Table 3). The pain was continuous since onset in all the patients. However, only 33 % patients were able to recall the exact day their headache started. There were no statistically significant differences in any parameter between the groups except the migrainous features (which were the differentiating points between the groups) (Table 1). Side-locked pain, nausea, vomiting, photophobia, and phonophobia were more in NDPH-mf. These all symptoms were statistically significant. Side-locked migraine, hemicrania continua, and other strictly unilateral headaches were excluded carefully in patients with unilateral head pain. All patients with unilateral headache (11 patients) received an indomethacin trial. None of the patients showed complete or marked response to indomethacin. Although we could not rule out the possibility of indomethacin resistant headaches, it seems less likely as typical exacerbations and autonomic features were not noted in these patients.

All patients had daily headaches. However, Seven patients (11 %) (five patients in NDPH-ICHD and two in ICHD-mf groups) had pain-free period in a day on a few occasions in a month. All patients had non-throbbing base line pain. More than 50 % patients had throbbing headaches during the exacerbations. Throbbing pain during the exacerbation was more in NDPH-mf than NDPH-ICHD (64 vs. 54 %). However, it was statistically not significant. Aggravation by physical activities was also more common in NDPH-mf than NDPH-ICHD (32 vs. 24 %) (statistically non-significant). Overall, 14 % patients reported cranial autonomic feature (either conjunctival injection or tearing), with slight preponderance in NDPH-mf group (23 vs. 9 %). None of the patients reported auras.

### Triggers

Overall, 54 % patients remembered a trigger, without any differences between NDPH-mf and NDPH-ICHD (55 vs. 54 %). The most common trigger was infection (especially respiratory tract infection). Overall, 29 % patients correlated the onset of headache to the infection. Injury, stress, and surgery were other triggers. Injury (mainly fall) was reported as a trigger by seven (11 %) patients. Three patients had fallen from height. Two patients had slipped down while walking down a stairs. Another two patients were assaulted manually. Three of them had mild head trauma. As the injury was mild, none of them was investigated for the injury.

### Prior history of headaches

A past history of headache was noted in 54 % patients (29 % tension-type headache and 25 % migraine). No patient had a past history of chronic daily headache (CDH) or escalation of headache frequency prior to the onset of NDPH. We compared NDPH with a past history headache to NDPH with no such history of headache (Table 2). Migrainous features (such as throbbing headache, nausea, vomiting, photophobia, phonophobia, and aggravation by physical activities) were more common in NDPH with a past history of migraine. Cranial autonomic features were also more common in patients with NDPS-mf (25 vs. 6 %). However, none of these features were statistically significant.

**Table 2:** Comparison of clinical features of NDPH (Kung et al.’s) in patients with and without past history of headache

	NDPH (with past history of TTH) (*n* = 18)	NDPH (with past history of migraine) (*n* = 16)	NDPH (with past history of no headache) (*n* = 29)
Gender (female)	12 (67 %)	9 (56 %)	15 (52 %)
Duration of illness			
Mean (SD)	27.2 ± 20.1 months	29.0 ± 18.8 months	28.0 ± 20.3 months
Range (months)	3–72	4–60	4–72
Unilateral pain	5 (28)	2 (13)	4 (14)
Type of pain			
Non-throbbing	18 (100)	16 (100)	29 (100)
Throbbing	6 (33)	8 (50)	17 (59)
Aggravation by physical activities	3 (17)	4 (25)	3 (10)
Hampering routine activities	2 (11)	1 (6)	1 (3)
Associated features			
Nausea	8 (44)	12 (75)	10 (34)
Vomiting	0 (0)	1 (6)	3 (10)
Photophobia	6 (33)	7 (44)	9 (29)
Phonophobia	4 (22)	7 (44)	6 (21)
Autonomic symptoms	1 (6)	4 (25)	3 (10)

*NDPH* new daily persistent headache according to second edition of the International Classification of Headache Disorders, *TTH* Tension type headache

### Investigations

The neurological and general examination (including fundoscopy) did not reveal any other abnormality in any patient. All patients underwent neuroimaging. Fifty-seven patients (90 %) had a brain MRI, and the remaining six patients (10 %) had a head CT scan. Of the 57 MRI brain, 34 patients (54 %) had contrast enhanced MRI scan. Ten patients (16 %) were subjected to magnetic resonance venography. Neuruoimaging studies were essentially normal or were not casually related in any patient. Fourteen patients (22 %) were subjected to lumbar punctures. All had normal opening pressure. Cerebrospinal fluid (CSF) examinations were normal except the presence of mild pleocytosis (<10 lymphocytes/mm^3^) in three patients and a slightly elevated protein (50 mg/dl) in one patient.

### Treatment and follow-up

As there are no guidelines for the management in patients with NDPH, treatment was not standardized. All patients received medications. The treatment for most patients was a combination of various drugs. The most preferred treatment was a combination of steroid (intravenous methyl prednisolone followed by oral therapy) + sodium valproate (intravenous followed by oral) + antidepressant (amitriptyline or dothiepin) ± naprosyn (250–500 b.i.d). Steroid was given in the form of intravenous methyl prednisolone (IV MPS) (500–1,000 mg daily for 3–5 days), followed by oral steroid (prednisolone 1 mg/kg body wt) for 7–10 days. Sodium valproate was initially given in the intravenous form at the dose 15 mg/kg body weight (loading), followed by 5 mg/kg 8 hourly for 3–5 days. Intravenous sodium valproate was followed by oral valproate (500–1,500 mg/daily) for 3–12 months (depending on the patients’ symptoms). Antidepressant was also given for 3–12 months (amitriptyline 25–75 mg/daily or dothiepin 25–75 m/daily). Naprosyn was given at the dose of 250–500 mg b.i.d for 1–3 weeks. This combination of the drugs was based on our clinical experiences and the suggested treatment for the other chronic daily headaches in the literature. Other commonly used drugs were topiramate, propranolol, flunarizine and leviteracetam. The duration of the treatment for the patients with CDH is not well defined. Doddick [10] suggests that medication should be continued for at least 3–6 months until the patient gets a satisfactory response. We follow this recommendation and gave therapy for at least 3–6 months after achieving the target response. The response rate to the drugs was calculated at the end of the follow-up. The mean follow-up period was 9.3 ± 4.6 months. Earlier we reported nine patients with post-infectious headache of shorter duration (6–20 weeks) who showed a response to intravenous methyl prednisolone [11]. However, only four had headache duration of >3 months. In order to assess the treatment, we divided the patients into three groups: (1) NDPH with 3–6 months of duration, (2) NDPH with >6–24 months duration, and (3) NDPH with >24 months duration (Table 3). Overall, 23 patients (37 %) showed an excellent response. The excellent response was highest (58 %) in patients with NDPH of 3- to 6-months duration. However, only 26 % with NDPH of more than 2-years duration showed a complete response. Overall, nine patients (14 %) still had daily persistent headache. Six of the 27 patients (22 %) with NDPH of >2-years duration had poor response to drugs (minimal or no response). 83 % patients with a history of less than 6-months durations had either Excellent or Good prognosis. However, none of these data were statistically significant in comparison to other groups.

**Table 3:** Therapeutic responses according to the duration of the illness

	NDPH (All patients) (*n* = 63) (%)	NDPH (3–6 months) (*n* = 12)	NDPH (>6–24 months) (*n* = 24)	NDPH (>24 months) (*n* = 27)	*p* value
Excellent (no or less than one headache per month), *n* (%)	23 (37 %)	7 (58 %)	9 (38 %)	7 (26 %)	0.16
Good (>50 % reduction in headache frequency or days per month) *n* (%)	19 (30 %)	3 (25 %)	7 (29 %)	9 (33 %)	0.87
Fair (<50 % reduction in headache frequency or days per month)	12 (19 %)	2 (17 %)	5 (21 %)	5 (19 %)	1.0
Poor (Minimal or no response).	9 (14 %)	0 (0 %)	3 (12 %)	6 (22 %)	0.23

The combination of IV MPS + intravenous sodium valproate + antidepressant (amitriptyline or dothiepin) ± naprosyn was given to 37 patients. The clinical responses in these 37 patients were: Excellent, 17 (46 %); Good, 11 (30 %); Fair, 6 (16 %); and poor response, 3 patients (8 %). We compared the treatment response of the patients receiving this combination to the patients who did not receive this combination (data not shown). Although, none of the values reaches the statistically significant, the response was more favorable in the patients receiving combination therapy (46 vs. 19 % for excellent response). Response to the drugs started on 2–5th day of the intravenous therapy and maximum improvement was achieved in 2–6 weeks. A few patients (10 patients) received cycle of IV MPS on two or more occasions (nine patients: 2 times; one patient: 3 times). Four patients received another cycle because of poor response after first therapy (after 2–3 months). There was improvement in headache symptoms in three patients (grade of improvement changed from poor response to “Good” response). Another two patients received second cycle of IV MPS because of the incomplete response (both showed improvement). Four patients received second cycle because of the recurrence of symptoms. These all patients showed “excellent” response to second cycle.

No serious side effects were noted. Two patients complained about Cushingoid symptoms (especially facial edema). Two patients had pain abdomen. Three patients developed leucocytosis that returned to normal in 7–10 days. All patients had normal blood pressures during the hospitalization for the intravenous therapy.

Medication overuse was noted in eight patients. Five out of these eight patients had headache of >2-years duration. Other five patients had headache of 6- to 24-months duration. The responses to treatment in these patients were: Excellent, 2; Good, 3; Fair, 1; and poor response, 2 patients.

We also compared the treatment response in patients having triggers to that of without triggers (Table 4). Patients with a recognized trigger showed better prognosis. Excellent response was about two times higher in patients with a known trigger (47 vs. 24 %). However, it was statistically not significant (*p* value 0.0714). Sixteen patients (out of 34) with a known trigger showed excellent response. The patients with a preceding history of infection showed more favorable response. There were 18 patients with the preceding history of infections, 12 of them (67 %) showed excellent response.

**Table 4:** Therapeutic responses in NDPH patients with recognized triggers and with no triggers

	NDPH with No trigger factors *n* = 29	NDPH with trigger factors *n* = 34	*p* value
Excellent, *n* (%)	7 (24 %)	16 (47 %)	0.0714
Good, *n* (%)	10 (34 %)	9 (26 %)	0.5855
Fair, *n* (%)	6 (21 %)	6 (18 %)	1.0
Poor, *n* (%)	6 (21 %)	3 (9 %)	0.2804

## Discussion

The present diagnostic criteria of NDPH (of ICHD-II) exclude the prominent migrainous features in NDPH. There were very few case series of NDPH before the introduction of ICHD criteria of NDPH [1, 7, 12]. However, these all case series had patients with prominent migrainous features. Even after the development of ICHD criteria for NDPH, most authors bypassed it and used broader set of criteria which includes migrainous features [2, 3, 6, 8, 9, 11]. In this retrospective study, we examined the patients fulfilling the Kung et al.’s criteria for NDPH, which allows to include migrainous features. Our study is probably the third largest case series on NDPH (after Robin et al.’s and Peng et al.’s observations).

In our cohort, about two-thirds of patients (65 %) fulfilled the IHS criteria for NDPH. Twenty-two patients (35 %) had prominent migrainous features (NDPH-mf). In similar observation in other larger cohorts, 34–44 % patients had fulfilled the criteria for NDPH-ICHD [2, 3, 9]. Epidemiological and clinical features of these two groups were substantially similar, except the presence of migrainous features in NDPH-mf, which were the differentiating features between two. There were statistically significant differences for unilateral pain, nausea, vomiting, photophobia, and phonophobia.

Rozen [5] reviewed the literature for the presence of migrainous features in patients with NDPH. The prevalence for nausea in patients with NDPH was 33–68 %. Our 49 % patients reported nausea. Takese et al. [13] used the strict ICHD criteria to include NDPH patients and in that series photophobia was noted by only 3 % patients, while none of the patients had phonophobia. However, in other series both photophobia and phonophobia were common (photophobia 27–66 % and phonophobia 17–61 %). The prevalence of photophobia (33 %) and phonophobia (19 %) in our case series was toward the lower side of the existing range.

In original description of NDPH (Vanast case series) [1], patients did not have a prior history of headache. However, most other series on NDPH had patients with a prior history of headache. Robbins et al. [3] included only those patients who had headache frequency of <4 per month. In Peng et al. series [2], headache frequency was ≤1/month. In our observation, more than half (54 %) had a history of episodic headache (≤1/month). Comparison between patients with NDPH with past history of episodic tension-type headache and NDPH with past history of migraine was done (Table 2). Migrainous features and cranial autonomic features were more common in NDPH with the past history of migraine. Although, none of these features were statistically significant, such observations (high migrainous features and cranial autonomic features in patients with a past history of episodic migraine) have not been observed previously in any case series. This observation indicates that NDPH patients with a past history of episodic migraine may have more migrainous features. There may be several explanations for this association. As migrainous features may be the part of NDPH, this co-association is a normal phenomenon. This might also be because of superimposed attacks of migraine attacks in these patients. Although, no patients had escalation of frequency of migraine just prior to NDPH onset, we cannot rule out the possibility of abrupt transition of episodic migraine into chronic migraine in these patients, as about 20–30 % patients with CDH may have history of abrupt transition from episodic migraine into chronic migraine [12, 14]. There is no guideline to differentiate NDPH with migrainous features to chronic (daily) migraine with a history of abrupt transition from episodic headache to chronic headache.

We reviewed the literature to delineate the interrelation between the prior history of headache and migrainous features (nausea, photophobia, phonophobia). Most studies included migrainous features in their criteria. Prior history of headaches was noted between 25 and 38 % [2, 3, 7, 8] in these case series. Only one large series strictly followed ICHD criteria (excluding migrainous features) [13]. Nausea was reported by 33 % patients. However, photophobia was noted by only 3 % patients. None of the patients had phonophobia. The prior history of headache was noted in only 7 % patients in that series. Therefore, a possibility exists that a patient with a past history of episodic headache might have more migrainous symptoms. Moreover, patients with a past history of episodic migraine might have attacks of migraine even on the background of NDPH. This suggests that the presence of prominent migrainous features should be judged cautiously in patients with a past history of episodic headache (especially migraine).

### Therapeutic responses and prognosis

The first description of NDPH considered this entity as a “benign or self-limiting” form of headache [1]; however, most other observations considered it as the most refractory headache disorder. A review of the literature suggests that most studies were done on the patients who had headache duration of more than 6 months. It may be the reason for getting more refractory form of NDPH [15]. It is observed that CDH with daily pain is more treatment refractory than CDH without daily pain [16]. Patients with NDPH have ‘daily and continuous’ headache from the onset. Therefore, a possibility to become refractory to treatment is more with NDPH than any other CDH. A recent study done on patients with shorter duration (median 5 months) had demonstrated a relatively good prognosis [2]. Mean duration of illness in our case series was 27.5 months. However, our patients showed favorable out come. 37 % patients showed “excellent” response (no or less than 1 headache per month). Another 30 % patients had “good” response (>50 % reduction in headache frequency or days per month). Only 14 % patients had poor response. We categorized the patient in three groups to assess the response to treatment: NDPH (3–6 months), NDPH (>6–24 months), and NDPH (>24 months). We compared the response to treatment between the groups. Although, none of the values reach the statistical significance, patients with shorter duration (at the time of first visit) had better outcome. Excellent response was more than two times higher in patients with a history of less than 6 months in comparison to patients with history of >2 years (58 vs. 26 %). Although, a possibility of self-limiting form of NDPH cannot be ruled out in patients with NDPH of shorter duration (<6 months), our observations indicate that intervention in early stage could prevent chronification. However, a number of other explanations are also plausible. A possibility of self-limiting form is more in patients with headache of shorter duration. In the same line, chance of being refractory to the treatment is more in patients with history of longer duration. These all may be reasons for the good responses in patients with shorter duration.

There is no specific treatment strategy for NDPH [5, 17]. On this issue Rozan [5] writes “Most headache specialists will treat NDPH with the same acute and preventive medications that they use to treat chronic migraine….” Unfortunately, there is no formal evidence-based recommendation for optimal therapy in patients with CDH. Most published guidelines are the personal experience of the authors or the summary of the available evidences. Probably it is the reason for heterogeneity in treatment of patients with NDPH in most case series (including ours).

A few authors suggest that aggressive intravenous therapy for intractable CDH is more cost- and time-effective mode of treatment [18]. Krusz suggests that intravenous therapy may help in breaking a long, unremitting cycle of CDH. Intravenous sodium valproate is one of the safest intravenous drugs used for the various headache disorders and its effects have been demonstrated in both intractable acute attack and chronic daily headaches [19–21]. In one open-label study, intravenous valproate was effective in a number of primary headache disorders (including CDH) [21]. Improvement in headache was noted by 80 % patients with CDH by intravenous valproate in another open-label study [20].

Various studies have demonstrated the beneficial effects of injectable steroids on acute intractable attack of migraine and other headache disorders. Anecdotal evidences suggest that intravenous steroid for the short term may effective even in CDH patients [17, 18]. A recent meta-analysis suggests that addition of steroid to the standard therapy for the management of acute migraine headache may decrease the incidence of the recurrence of headaches [22]. The dose and duration of steroids are highly variable. Most authors used steroids for a few days to about 2 weeks. Bonuccelli et al. [23] gave injectable dexamethasone (with amitriptyline) for 2 weeks in patients with CDH. Trucco et al. [24] gave intravenous dexamethasone (8 mg daily) (with other drugs) for 7–15 days. The authors [24] suggest that a higher dose or intravenous route (of steroids) may be required for patients with CDH/medication overused headaches.

Intravenous MPS is generally considered as safe as majorities of the side effects are transient and self-limiting and do not require specific treatment [25].

Our observation showed a lower percentage of medication overuse (13 %). This observation was similar to that of Kung et al.’s case series [6] where medication overuse was noted in only in 8.7 % patients. However, a few recent observations reported higher proportion of medication overuse in patients with NDPH. Robbins et al.’s [3] reported medication overuse in about 45 % patients. In Peng et al.’s series [2], medication overuse was noted in about 35 % patients with NDPH. The factors predicting development of the medication overused headache have not been explored in the literature. However, the presence of such wide variability of prevalence of medication overuse headache in patients with NDPH suggests multifactorial involvement.

Chronic daily headache has been linked to various comorbid psychiatric conditions. Associated psychiatry disorders can lead to poor response to therapy. However, there are very few studies in patients with NDPH in which psychiatric evaluation was performed. Robbins et al.’s reported self-reported anxiety and self-reported depression in about one-third of the patients. In Peng et al. case series [2], psychiatry comorbidities were noted in more than half of the patients. However, in both case series, associated psychiatric comorbidities were not related with the outcomes. Detail psychiatric evaluations were not done in our patients. Self-reported depression and anxiety were noted by a few patients in our case series (19 and 16 % respectively) (Table 1). No association was noted between clinical outcomes and self-reported depression and anxiety. However, as comorbid psychiatry disorders are known to have a poor response to therapy, better outcome in our case series may be because of low rates of comorbid depression and anxiety.

The etiology of NDPH is poorly defined. About 40–60 % patients recognize triggers at the onset of the headache [2, 3, 5, 12]. Our 56 % patients had recognized triggers at the onset of NDPH. Besides infectious etiology (29 %), other triggers in our patients were injury, stress, surgery, and post partum state. Injury was reported as a trigger by seven (11 %) patients. Three patients had mild head trauma. A possibility of chronic post-traumatic headache attributed to mild head injury (ICHD-II code 5.2.2) [4] exists in these patients. However, it was less likely as none of the patients had symptoms and/or signs suggestive of concussion (a must to fulfill the criteria of 5.2.2) [4]. Although most case series did not report head trauma as a trigger, it was the second most common inciting factor (23 %) in Mack’s series [12]. Surgery and stress are two important triggers for the development of NDPH. We think injury or trauma may be analogous to surgery. Moreover, injury itself may produce stress (another trigger for NDPH). Most case series excludes the injury as triggers for NDPH. However, our case series and Mack’s observation suggest that injury may be a trigger for the development of NDPH, and mechanisms responsible for it may be entirely different from that of post-traumatic headache. Though statistically not significant, patients with recognized triggers showed better outcome. A study with larger sample of patients is required to confirm the observations.

Ethnic and geographical variation should also be taken into consideration for such type of observation, especially for post infectious variant, as infectious agents vary with the environmental factors. All patients received drugs before reporting to us. Therefore, we cannot rule out a possibility of delayed response of previously used drugs in a few patients. Therefore, our observations should be judged very cautiously.

### Limitation of the study

It is a retrospective study and possibilities of unrecognized selection bias and recall bias exist. There was a low recall of the headache onset day compared to other NDPH series. Therefore, a possibility of recall bias about onset of headache may be high in our case series. In addition, headache management and treatment were not standardized. Besides these, we cannot rule out even the possibility of other cause of headache (secondary), as full evaluation for secondary headache was not done (contrast enhanced MRI and MRV were not done in each patient). The patients in our study were seen in an adult tertiary neurology clinic. Therefore, our observations cannot be generalized as our sample of patients may not truly represent patients with new onset headaches due to referral and other biases. Despite a number of limitations in the study, the positive outcome suggests that further research is necessary.

## Conclusion

Migrainous features might be the common features in patients with NDPH, however, its presence should be judged cautiously in patients with past history of episodic headache, especially episodic migraine. Our observations hint that early intravenous therapy may prevent chronification. However, we would not like to draw any conclusion as it was a retrospective, open-label, and uncontrolled study.

*Consent* Written informed consent was taken from the patients to publish the report.
